# Prior Outpatient Care Use in Emergency Department Patients with Low- and High-acuity Conditions in Germany

**DOI:** 10.5811/westjem.38466

**Published:** 2025-09-20

**Authors:** Yves N. Wu, Martin Möckel, Dörte Huscher, Antje Fischer-Rosinský, Thomas Keil, Anna Slagman

**Affiliations:** * Charité - Universitätsmedizin Berlin, Emergency and Acute Medicine (CVK, CCM), Berlin, Germany; † Charité - Universitätsmedizin Berlin, Institute of Medical Biometry and Clinical Epidemiology, Berlin, Germany; ‡ Charité - Universitätsmedizin Berlin, Institute of Social Medicine, Epidemiology and Health Economics, Berlin, Germany; § Institute of Clinical Epidemiology and Biometry, University of Würzburg, Würzburg, Germany; || State Institute of Health I, Bavarian Health and Food Safety Authority, Erlangen, Germany

## Abstract

**Introduction:**

The key role of emergency departments (ED) is to treat severe and life-threatening cases. A rise in ED visits, particularly for low-acuity conditions, places a burden on resources which may hinder efficient care for high-acuity conditions. We investigated the association between previous outpatient healthcare services use and low-acuity visits in EDs in Germany.

**Methods:**

We analyzed data from the Initiative for Emergency Department Evaluation and Data Collection project, with 454,747 ED visits by 353,926 patients collected from 16 EDs in Germany in 2016. We included a subset of 228,753 (64.6%) patients with 299,914 (66.0%) visits from 12 of the participating EDs for which outpatient care data was available. We categorized ED presentations into low- or high-acuity based on transportation to the ED, triage category, hospital admission status, and intrahospital mortality. By merging patient hospital records with outpatient billing information, we assessed outpatient care utilization prior to ED visits. Using a generalized mixed-effects model, we investigated the relationship between acuity level and outpatient care utilization, adjusting for age, sex, and type of residential area.

**Results:**

Low-acuity patients were considerably younger than high-acuity (mean age ± standard deviation: 45 ±19 vs 58 ±21 years) and used outpatient services less often within 10 days prior to their ED visit: 40.6% vs 49.5%. Key associations for low-acuity ED visits included younger age (per 10-year categories: adjusted odds ratios 0.72, 95% confidence interval 0.72–0.73), urban residence (1.17; 1.13–1.22), and timing of the last outpatient contact. Longer durations since the last outpatient contact were associated with a higher likelihood of presenting to the ED with low-acuity symptoms. Compared to patients who visited their primary care physician (PCP) shortly before their ED visit, those with PCP contact 1–6 months (1.22; 1.19–1.25) and over six months prior (1.33; 1.26–1.41) were more likely to present with low-acuity conditions.

**Conclusion:**

While almost half of both low- and high-acuity patient groups utilized outpatient services prior to the ED visit, low-acuity patients were generally younger and had fewer such contacts. The majority had accessed both primary care and the ED, challenging the assumption that low-acuity patients routinely bypass outpatient care before seeking emergency services. This raises the question of what limitations or unaddressed needs in outpatient care drive these patients to seek subsequent care in the ED. More research is needed to explore the structural and systemic factors influencing low-acuity ED visits.

## BACKGROUND

Emergency departments (ED) are crucial components of healthcare systems, ideally designed to manage serious and life-threatening cases. However, studies have shown that a significant proportion of patients visiting EDs have conditions that might not necessarily warrant emergency care.^1^,^2^ The evolving role of emergency and urgent care systems as well as outpatient care availability has been linked to increased use of EDs, particularly outside regular hours of primary care physicians (PCP). This pattern can be attributed to various factors, including the accessibility of services and patient anxiety surrounding health issues, prompting individuals to seek immediate attention in highly equipped medical settings.^3^,^4^

While ensuring timely access to emergency care is vital, a high influx of non-emergency visits can often strain ED resources and may hinder the efficient delivery of care to patients with urgent needs, resulting in ED crowding.^5^–^7^ To alleviate this burden, it is crucial to broaden the scope of research beyond the boundaries of the ED, directing attention to the roles of healthcare services outside the emergency setting. Emphasis should be placed on outpatient services, such as PCPs, medical specialists, and telehealth services, which can be attributed a gatekeeper function to the ED. These services serve as pivotal entry points in directing traffic toward the ED and have a key role in regulating the distribution of resources and directing patients to the most suitable care setting.^8^–^10^

The German healthcare system is primarily funded through a dual insurance model, comprising statutory health insurance (GKV) and private health insurance (PKV).^11^ Approximately 88.1% of the population (73.3 million people) are insured under the GKV, while 10.5% (8.7 million) are covered by PKV, which can serve as both primary coverage for eligible individuals and supplemental insurance for those in the GKV seeking additional services. Additionally, Germany has a high PCP use rate, with 30% of the population visiting a doctor three to five times per year.^11^ However, what is currently lacking is a nationwide gatekeeping system for urgent care services, and the relationship between prior outpatient healthcare engagement and ED use remains largely unexplored.^12^–^13^ In this study we aimed to determine whether low-acuity ED patients differed from high-acuity patients in terms of the timing and frequency of outpatient visits in the period leading up to their ED visit. Specifically, we investigated whether low-acuity patients were less likely than high-acuity patients to engage with outpatient healthcare services before seeking emergency care, thereby assessing the validity of the claim that low-acuity patients use the ED for convenience rather than as a last resort.

## METHODS

### Study Design and Setting

This study is based on data from the Initiative for Emergency Department Evaluation and Data Collection (INDEED) conducted across 16 German hospitals with EDs in 2016. Patients were eligible if they had at least one ED visit recorded in 2016 and were insured by a German statutory health insurance provider. To ensure eligibility for outpatient care data linkage, patients had to be at least 20 years of age on January 1, 2016, corresponding to a minimum age of 18 at the start of the observation period (January 1, 2014). Routine hospital data from all ED patients were merged with statutory health insurance records covering outpatient care from two years before to one year after the ED visit (2014–2017), resulting in a dataset of 454,747 ED visits from 353,926 patients. Due to missing data required for the primary outcome or outpatient utilization, only 12 of the 16 EDs were included in the final analysis.

**Population Health Research Capsule**

What do we already know about this issue?

*Low-acuity ED visits are common and often assumed to stem from poor outpatient care access, straining emergency services*.

What was the research question?

*Do low-acuity ED patients differ from high-acuity ones in prior outpatient-visit timing and frequency?*

What was the major finding of the study?

*Low-acuity visits were associated with fewer recent outpatient contacts (OR 1.22–1.33; 95% CI 1.19–1.41)*.

How does this improve population health?

*Findings suggest that EDs supplement, not replace, outpatient care, which could guide resource planning and patient flow improvements*.

### Study Population and Outcome Definition

To assess patient characteristics and prior outpatient care utilization, ED visit data was merged with patient-level outpatient data. The outcome definition followed the pragmatic framework by Slagman et al (2023), which allows for a reliable and replicable identification of high- and low-acuity ED visits in German routine data. This approach incorporates objective criteria such as hospital admission, triage category, and transport type, reflecting their consistent availability and utility in routine emergency documentation. It is particularly suited to the German healthcare setting and facilitates standardization across sites and studies, despite the absence of a gold-standard definition of “acuity” in routine data.^2^ High-acuity visits were identified by fulfilling at least one of the following criteria:

1. admitted to hospital or deceased,
2. medically accompanied transport, or
3. Manchester Triage System (MTS) or Emergency Severity Index (ESI) triage category of 1, 2 or 3.

The MTS is a structured tool that categorizes ED patients into five levels (immediate, very urgent, urgent, standard, and non-urgent), each with a corresponding maximum waiting time.^14^ The ESI is also a five-level triage system. However, it prioritizes patients by acuity and anticipated resource needs, ranging from immediate life-saving intervention needed to non-urgent/no resources required.^15^ Cases that met none of these criteria were classified as low acuity. If the triage category was missing, the case was labeled as “not assessable.” Applying this framework to the INDEED dataset, we excluded 83,991 visits due to missing acuity data. Since the integration of ED and outpatient billing data was required to evaluate previous use patterns of outpatient healthcare services, another 57,214 patients (corresponding to 70,842 visits) were excluded due to missing outpatient records. Additionally, four study sites were excluded due to incomplete data, leaving a final study population of 299,914 visits from 228,753 patients across 12 EDs. We conducted a comparison of included and excluded cases to assess potential selection bias (Appendix Table 2).

### Definition of Influential Variables

We determined baseline patient characteristics using district code numbers to identify residential districts, cross-referenced with the BBSR (Bundesinstitut für Bau-, Stadt- und Raumforschung, German Federal Institute for Research on Building, Urban Affairs, and Spatial Development) INKAR (Indicators and Maps for Spatial and Urban Development) database, which provides detailed development indicators for Germany.^16^ Based on population density, districts were classified as follows: urban (>300 inhabitants per square kilometer [i/km^2^]); regions with signs of urbanization (150–300 i/km^2^); or rural (<150 i/km^2^).^16^ We analyzed ED length of stay (LOS) only for non-admitted patients, as all inpatients were classified as high acuity. To ensure data reliability and avoid anomalies, a cutoff was defined, considering only cases within a range of 5 minutes to 24 hours (1,440 minutes).

### Data Management

To assess outpatient healthcare utilization, the analysis relied on timestamps from outpatient billing data as indicators of service use. To ensure accuracy, we excluded outpatient billing data originating from the participating EDs, as our aim was to assess outpatient healthcare utilization outside of the emergency department setting. Non-relevant services (e.g., antibody testing, postage fees, and laboratory tests) were also excluded. Lastly, outpatient contacts recorded on the same day as the ED visit were removed to prevent misclassification of ED services as outpatient care. We categorized PCP and specialist visits based on the last two digits of the German physician identification number, ensuring an accurate classification of outpatient services.^18^

### Statistical Methods

We conducted descriptive analyses to compare ED visit characteristics by acuity level. Chi-square tests assessed categorical variables (sex, region type, weekday, and prior outpatient contact), while Wilcoxon rank-sum tests were used for continuous variables (age and ED LOS) due to their non-normal distribution, as it was confirmed via the Kolmogorov-Smirnov test. A mixed-effects multivariable logistic regression model estimated the impact of key variables on acuity level (low/high). This binomial model, fitted by maximum likelihood (the Laplace approximation), included random effects for ED site and individual patients to account for non-independence of observations. Since the analysis was case level (ED visit), each visit was treated independently. And since the analysis was done on ED visit-level, each visit was treated independently. For patients with multiple outpatient visits, only the most recent contact before each ED visit was considered, allowing outpatient contact timeframes to vary across visits. This ensured that prior outpatient use was assigned specific to each ED visit, preventing bias from cumulative visit histories. The model adjusted for age, sex, region type, and prior outpatient use, with model selection based on Akaike Information Criterion (AIC) and Bayesian Information Criterion (BIC) to balance fit and complexity. We conducted analyses in R v4.3 (R Foundation for Statistical Computing, Vienna, Austria) using the tidyverse and lme4 packages.^20^–^21^

### Adherence to Retrospective Review Studies criteria

This study adhered to the methodological standards outlined by Worster et al (2005) for retrospective chart reviews in emergency medicine research. These criteria address key methodological aspects, including case selection, data abstraction, variable definition, missing data management, and quality control measures.^22^ A detailed listing of each criterion and its application within this study can be found in Appendix Table 1.

## RESULTS

**Figure 1:**
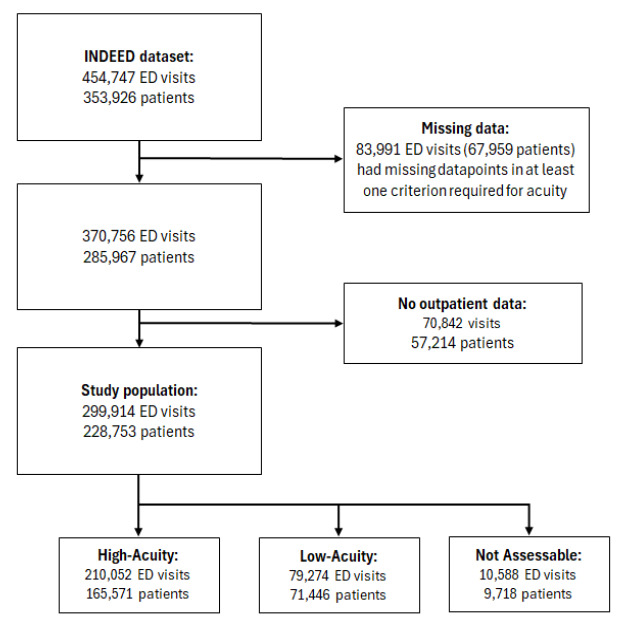
Selection of the study population from the INDEED* dataset. This flowchart outlines the exclusion process, where cases with missing acuity classification data or unavailable outpatient billing records were removed. Since patients can have multiple ED visits, the sum of patients across acuity groups exceeds the total study population. *INDEED, Inanspruchnahme und Versorgungsmuster von Patienten in Notfallversorgungsstrukturen in Deutschland (Utilisation and Cross-Sectoral Patterns of Care for Patients Admitted to Emergency Departments in Germany)**;** *ED*, emergency department.

The final study population included 228,753 individual patients and 299,914 ED visits, representing 64.6% of the total population and 66.0% of all visits. Of these visits, 70.0% were presentations with high-acuity and 26.4% with low-acuity conditions. For 3.5%, the acuity level was not assessed due to a missing triage score (Figure 1).

As seen in Table 1, patients with ED visits classified as low acuity were, on average, 13 years younger than those with high-acuity visits. Both high- and low-acuity cases were similarly distributed across rural and urban areas, with urban regions accounting for most cases in each category. Although most visits took place on weekdays, low-acuity visits were more frequent during weekends compared to those of high acuity. The LOS in the ED varied considerably between both groups, with the median ED stay for low-acuity visits being significantly shorter than for high-acuity visits. Since many high-acuity cases were admitted as inpatients, only 35% of these visits were included, whereas we evaluated all low-acuity cases with available data on LOS (77.5%).

**Figure 2:**
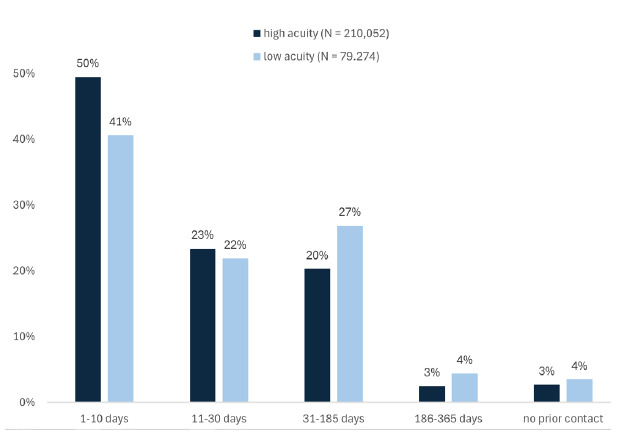
Bar chart displaying last outpatient contact within one year before emergency department (ED) visit, stratified by acuity level, displaying the percentage of high-acuity (dark blue) and low-acuity (light blue) ED patients who had their last outpatient contact within the timeframes 1–10 days, 11–30 days, 31–185 days, 186–365 days, or no contact at all prior to their ED visit from data based on 12 German hospitals included in the INDEED* dataset. *INDEED, Initiative for Emergency Department Evaluation and Data Collection.

While most ED visits (82.0%) were from patients with 1–2 visits, high-acuity presentations were more common among frequent ED users. Among visits from patients with 3–9 ED presentations, 18.2% were high acuity compared to 13.2% low acuity. Very frequent ED users (≥10 visits) accounted for only 1.1% of all visits across both acuity groups. We assessed outpatient care use, focusing on the percentage of patients who visited outpatient healthcare services in general, a PCP, or specialist before their ED visit. Low-acuity patients were less likely than high-acuity patients to have had an outpatient contact within 10 or 11–30 days before their ED visit. This pattern was seen for both PCP and specialist visits. Furthermore, a higher proportion of low-acuity patients had their last outpatient visit over six months before their ED visit.To assess outpatient care use prior to an ED visit, we analysed the most recent contact within specific timeframes before the ED visit. We found that both groups demonstrated substantial engagement with outpatient healthcare services before their visit to the ED, while only a small proportion had no recorded contact within the previous 12 months. However, high-acuity patients were more likely to have had their last outpatient visit in a more recent timeframe compared to low-acuity patients. This difference was considerably larger within 10 days before the ED visit (Figure 2).

Based on these results, we performed a mixed-effects regression analysis to further study the variables of interest. Prior to regression analysis, the intraclass correlation coefficient (ICC) was estimated using a variance components approach, where the ICC represents the proportion of variance attributable to differences between ED sites. The ICC for the random effect study site on the outcome low- or high-acuity was estimated at 0.023 (95% CI 0.012–0.064). As depicted in Table 2, crude regressions, along with mixed-effects regression models that incorporated study site and patient as random effects were performed to assess the influence of outpatient attributes on low-acuity ED visits, while adjusting for sex, age, and region type. Each attribute was assigned to a mixed-effects model, with model 1 assessing last outpatient contact in general, model 2 last PCP contact, and model 3 last specialist contact of patients before their ED visit..Across all three models female patients (odds ratio [OR] 1.05) and those living in urban areas (OR 1.18–1.19) had a slightly higher likelihood of a low-acuity ED visit. In contrast, the likelihood decreased with increasing age (OR 0.72, per decade) and for patients in mixed urban-rural areas compared to those in rural areas (OR 0.85–0.87). Patients with a higher number of ED visits had lower odds of presenting to the ED with low-acuity conditions. Compared to those with 1–2 ED visits, patients with 3–9 visits had 20% lower odds of a low-acuity visit (OR 0.80), while those with ≥10 visits had 14–17% lower odds (OR 0.82–0.86). The odds of a low-acuity ED visit increased with increasing time since the last outpatient contact, regardless of the type of contact. Compared to patients who had outpatient contact within 10 days before their ED visit, those with contact 11–30 days prior had slightly higher odds of a low-acuity ED visit (OR 1.09–1.10). The odds further increased for those with contact 1–6 months prior (ORs 1.20–1.22) and six months to a year prior (OR 1.30–1.37). Having no prior outpatient or specialist contact in the past year was associated with slightly increased odds of a low-acuity ED visit (OR 1.06–1.07, Table 2). The Akaike Information Criterion (AIC) and Bayesian Information Criterion (BIC) values were comparable across all three models, with Model 1 showing the highest values and Model 2 the lowest, which suggests that Model 2 provides the best fit (Table 2).

## DISCUSSION

### Summary of Main Results

In this study of 299,914 ED presentations from 228,753 patients in German EDs in 2016, we found that patients with low-acuity conditions were, on average, younger than those with high-acuity conditions. While a large proportion of low-acuity patients had engaged with outpatient healthcare services within a year before their ED visit, their use was slightly lower compared to high-acuity patients. Results from the multivariable mixed-effects model confirmed that age, region type, and prior healthcare use were associated with low-acuity ED presentations. Patients with recent outpatient, PCP, or specialist contact before the ED visit were less likely to present with low-acuity conditions. Additionally, frequent ED users had a lower probability of low-acuity visits, suggesting that repeated visits are more often associated with high-acuity cases. Among the models tested, the mixed-effects model incorporating prior PCP contact had the lowest AIC and BIC values, indicating a better balance between model fit and complexity, suggesting it to be the most generalizable for understanding prior outpatient engagement in ED patients.

### Comparability of Findings

The findings of this study provide further understanding of who presents to the ED with low-acuity symptoms and why. While previous studies have emphasized lack of accessibility as a key driver of ED attendance, the results of this study show extensive engagement of both high- and low-acuity patients with outpatient healthcare services before their ED visit.^6^,^23^–^25^ This suggests that the ED is not always the first point of contact for many patients and that some patients seek ED care for conditions that may not have been satisfactorily treated or had worsened following outpatient care. Beyond accessibility, patient awareness and decision-making processes also play a crucial role in ED use. Kümpel et al (2024) highlighted that limited awareness of alternative care options contributes to ED visits for non-urgent conditions, even when outpatient services are available.^26^ Similarly, a US-based study by Heinert et al (2020) found that a lack of awareness of nearby alternative care facilities was common among ED users, suggesting that educational interventions could help lower non-emergent ED visits.^27^ Furthermore, insurance status significantly influenced ED use, with Medicaid, Medicare, and uninsured patients disproportionately choosing the ED over urgent care centers.^27^

Despite having more limited access to outpatient healthcare services, patients from rural areas were slightly more represented in high- compared to low-acuity visits (19% vs 17%). This was further underlined by the mixed-effects regression models, which showed that residing in urban areas was associated with a higher likelihood of a low-acuity ED visit. These findings mirror the results of Schmiedhofer et al (2016), who found that patients from rural areas were more strongly connected to a PCP, whereas urban patients were often loosely connected or did not have a PCP.^4^ Given that urban areas typically have a higher density of outpatient services, the expansion of these services alone may not be sufficient to reduce ED crowding due to low-acuity visits.^28^–^29^

A combination of factors—including accessibility, personal beliefs, emotional distress, and knowledge about alternative care options—likely contributes to the decision to seek ED care.^1^, ^4^, ^26^, ^27^, ^30^ Aside from accessibility and geographic factors, the results also align with previous studies regarding age-related trends in ED use. Younger patients had a higher likelihood of presenting to the ED with low-acuity symptoms, a finding consistent with prior research.^31^–^33^ This is likely due to the higher prevalence of complex and chronic conditions among older adults, making high-acuity ED visits more common in this group.^6^, ^31^–^33^ However, the specific reasons why younger individuals frequently visit the ED for low-acuity conditions remain unclear. Based on international literature, potential explanations include a greater reliance on emergency services among young adults and an increasing number of psychiatric emergencies in this demographic.^35^, ^36^ Further research on low-acuity ED presentations among young adults should aim to explore these assumptions.

Efforts to reduce low-acuity visits have explored alternative care models, such as walk-in clinics (WIC). Kurian et al (2022) found that introducing a WIC led to an immediate decline in low-acuity visits, suggesting that providing accessible alternatives can temporarily divert non-emergent cases from EDs.^37^ However, this effect was not sustained over time, indicating that WICs alone are insufficient to change patient behavior long term.^37^ To create a lasting reduction, additional strategies, such as expanding patient awareness of alternative care options, removing barriers to outpatient care accessibility, and introducing referral mechanisms, may be required.

## STRENGTHS AND LIMITATIONS

The strengths of the present study included the large sample of participating EDs across Germany and the unique dataset for which we merged routine hospital data from 2016 with billing data from statutory health insurance companies from 2014–2017 on an individual patient basis. However, several limitations should be noted. First, there is the possibility of data loss due to the use of retrospective data obtained from different EDs, which involved different hospital information systems, and thus varying data collection processes between study centers. This led to a high amount of missing data, which in turn had the potential to introduce bias. Most importantly, the dataset was constrained by the information available from participating EDs, and potentially relevant data points could not be collected in a standardized manner.

Secondly, because only statutory health insurance data was available, it remains unclear whether results would be the same for the ≈10% of patients in Germany with private health insurance.^11^, ^38^ Third, since the participating hospitals were not selected randomly, these results cannot be considered representative for the whole country. However, the 16 hospitals are in different regions across Germany and can be considered representative for their catchment area. Although the INDEED project presents the first approach in providing cross-sectoral ED and outpatient data across Germany, more effort is required to standardize multicenter data availability to enable a comprehensive understanding of potential factors that contribute to low-acuity ED use across large geographical settings. An example in this direction is demonstrated by the German *Emergency Department Data Registry*, AKTIN, which has proven to be applicable even in low-resource settings with restricted system access to facilitates monitoring processes in EDs across Germany.^39^

## CONCLUSION

This study showed that ED use, specifically for low-acuity patients, does not replace but rather complements prior outpatient care. This was reflected by the high outpatient use rates among both groups shortly before the ED visit. Additional research would be beneficial to discern whether these complementary visits are due to unmet patient needs, referrals from the outpatient sector due to resource constraints, or other factors.

## Supplementary Information



## Figures and Tables

**Table 1:** Characteristics of adult emergency department (ED) visits in Germany in 2016, stratified by acuity level, presenting demographic characteristics, ED visit details, and prior outpatient healthcare utilization. Visits are categorized as high acuity, low acuity, or not assessable based on predefined criteria. Variables include age, sex, region of residence, ED presentation time, length of stay, and engagement with outpatient healthcare services before the ED visit.

	High-acuity ED visits n = 210,052	Low-acuity ED visits n = 79,274	P-value	Unspecified ED visits n = 10,588	Total N = 299,914
Demographics					
Male	102,295 (48.7%)	38,172 (48.2%)	<.001	4,817 (45.5%)	145,284 (48.2%)
Age, years (mean [SD])	58 (20.9)	45 (18.7)	<.001	49 (20.4)	54 (21.1)
Region type			<.001		
Rural (<150 I./km^2^)	40,255 (19.2%)	13,488 (17.0%)		3,676 (34.7%)	57,419 (19.2%)
Signs of urbanization (150 – 300 I./km^2^)	26,975 (12.8%)	8,964 (11.3%)		506 (4,8%)	36,445 (12.2%)
Urban (>300 I./km^2^)	129,754 (61.8%)	50,519 (63.7%)		5,530 (52,2%)	185,803 (62.0%)
Missing	13,068 (6.2%)	6,303 (8.0%)		876 (8,3%)	20,247 (6.8%)
ED Visit					
Day of ED presentation			<.001		
Monday–Friday	146,614 (69.8%)	47,035 (59.3%)		6,524 (61.6%)	200,173 (66.7%)
Saturday / Sunday	63,026 (30.0%)	32,187 (40.6%)		2,623 (24.8%)	97,836 (32.6%)
Missing	412 (0.2%)	52 (0.1%)		1441 (13.6%)	1,905 (0.6%)
ED length of stay, minutes^1^			<.001		
Mean (SD)	199.9 (137.6)	164.6 (129.8)		163.7 (181.2)	183.1 (137.2)
Median	173	136		109	155
Interquartile range	105–264	77–217		51–207	89–243
NA or missing	136,535 (65.0%)	17,852 (22.5%)		5,565 (22.5%)	159,952 (53.3%)
ED visit frequency			<.001		
1–2 visits	169,597 (80.7%)	68,105 (85.9%)		8219 (77.6%)	245,921 (82.0%)
3–9 visits	38,128 (18.2%)	10,436 (13.2%)		2,054 (19.4%)	50,618 (16.9%)
≥10 visits	2,327 (1.1%)	733 (0.9%)		315 (3.0%)	3,375 (1.1%)
Outpatient care use					
Last outpatient contact before ED			<.001		
1–10 days	103,994 (49.5%)	32,212 (40.6%)		4,120 (38.9%)	140,326 (46.8%)
11–30 days	49,152 (23.4%)	17,360 (21.9%)		2,195 (20.7%)	68,707 (22.9%)
31–185 days	42,673 (20.3%)	21,326 (26.9%)		2,137 (20.2%)	66,136 (22.05%)
186–365 days	5,271 (2.5%)	3,470 (4.4%)		275 (2.6%)	9,016 (3.0%)
No prior contact	5,579 (2.7%)	2,810 (3.5%)		1,684 (15.9%)	10,073 (3.4%)
Last PCP contact before ED			<.001		
1–10 days	103,320 (49.2%)	31,986 (40.4%)		4,073 (38.5%)	139,379 (46.5%)
11–30 days	48,739 (23.2%)	17,218 (21.7%)		2,179 (20.6%)	68,136 (22.7%)
31–185 days	41,752 (19.9%)	20,924 (26.4%)		2,091 (19.8%)	64,767 (21.6%)
186–365 days	4,913 (2.3%)	3,270 (4.1%)		259 (2.5%)	8,442 (2.8%)
No prior contact	4,429 (2.1%)	2,064 (2.6%)		1,579 (14.9%)	8,072 (2.7%)
Last specialist contact before ED			<.001		
1–10 days	103,528 (49.3%)	32,109 (40.5%)		4,110 (38.8%)	139,747 (46.6%)
11–30 days	48,841 (23.3%)	17,272 (21.8%)		2,188 (20.7%)	68,301 (22.8%)
31–185 days	42,074 (20.0%)	21,038 (26.5%)		2,093 (19.8%)	65,205 (21.7%)
186–365 days	5,086 (2.4%)	3,346 (4.2%)		256 (2.4%)	8,688 (2.9%)
No prior contact	4,987 (2.4%)	2,496 (3.2%)		1,631 (15.4%)	9,114 (3.0%)

1 includes only non-admitted patients.

*PCP*, primary care physician; *I./km^2^*, inhabitants per square kilometer; *NA*, not applicable; *ED*, emergency department.

**Table 2:** Multivariable mixed-effects logistic regression models for low-acuity emergency department (ED) visits. Results of multivariable mixed-effects logistic regression models estimating odds of a low-acuity ED visit compared to a high-acuity visit. Crude and adjusted odds ratios with 95% confidence intervals are reported for demographic and healthcare utilization variables, including sex, age, region type, and timeframe of last outpatient, primary care physician, or specialist contact before the ED visit. Models adjust for sex, age, and region type, incorporating study site and patient-level random effects.

	Crude OR (95% CI)	Model 1		Model 2		Model 3	
		
		Adjusted OR (95% CI)	P-value	Adjusted OR (95%-CI)	P-value	Adjusted OR (95%-CI)	P-value
Sex, female	1.02 (1.01–1.04)	1.05 (1.03–1.08)	<.001	1.05 (1.03–1.08)	<.001	1.05 (1.03–1.08)	<.001
Age, per 10 years	0.73 (0.73–0.74)	0.72 (0.72–0.73)	<.001	0.72 (0.72–0.73)	<.001	0.73 (0.72–0.73)	<.001
Region type							
(ref.: <150 I./km^2^)							
150–300 I./km^2^	0.99 (0.96–1.02)	0.87 (0.82–0.92)	<.001	0.85 (0.80–0.90)	<.001	0.87 (0.83–0.92)	<.001
>300 I./km^2^	1.16 (1.14–1.19)	1.19 (1.15–1.23)	<.001	1.18 (1.14–1.22)	<.001	1.19 (1.15–1.23)	<.001
ED visit frequency							
(ref.: 1–2 visits)							
3–9 visits	0.68 (0.67–0.70)	0.80 (0.78–0.83)	<.001	0.80 (0.78–0.83)	<.001	0.80 (0.77–0.82)	<.001
>=10 visits	0.78 (0.72–0.85)	0.82 (0.72–0.94)	.01	0.86 (0.75–0.99)	.03	0.83 (0.72–0.95)	.01
Last outpatient contact							
(ref.: 1–10 days)							
11–30 days	1.26 (1.23–1.29)	1.09 (1.06–1.12)	<.001	-	-	-	-
31–185 days	1.76 (1.72–1.79)	1.20 (1.17–1.23)	<.001	-	-	-	-
186–365 days	2.22 (2.12–2.33)	1.30 (1.23–1.37)	<.001	-	-	-	-
No contact	1.77 (1.68–1.86)	1.06 (1.00–1.12)	.05	-	-	-	-
Last PCP contact							
(ref.: 1–10 days)							
11–30 days	1.14 (1.12–1.17)	-	-	1.09 (1.06–1.12)	<.001	-	-
31–185 days	1.62 (1.59–1.65)	-	-	1.20 (1.17–1.23)	<.001	-	-
186–365 days	2.15 (2.05–2.25)	-	-	1.30 (1.23–1.37)	<.001	-	-
No contact	1.51 (1.43–1.59)	-	-	0.96 (0.91–1.03)	.26	-	-
Last specialist contact							
(ref.: 1–10 days)							
11–30 days	1.14 (1.12–1.16)	-	-			1.09 (1.06–1.12)	<.001
31–185 days	1.61 (1.58–1.65)	-	-			1.20 (1.17–1.23)	<.001
186–365 days	2.12 (2.03–2.22)	-	-			1.30 (1.23–1.37)	<.001
no contact	1.61 (1.54–1.70)	-	-			1.06 (1.00–1.12)	.06
AIC	-	282428.8		276608.4		279309.9	
BIC	-	282565.2		276744.5		279446.1	

*AIC*, Akaike Information Criterion; *BIC*, Bayesian Information Criterion; *CI*, confidence interval; *PCP*, primary care physician; *I./km^2^*, inhabitants per square kilometre; *OR*, odds ratio; *ED*, emergency department.

## References

[1] Reinhold AK, Greiner F, Schirrmeister W (2021). Der Notfall „geht“ ins Krankenhaus: Eine Befragung von Patienten mit niedriger Dringlichkeit in einer Notfallaufnahme mit regionaler Alleinstellung. Med Klin Intensivmed Notfmed.

[2] Slagman A, Fischer-Rosinský A, Legg D (2023). Identification of low-acuity attendances in routine clinical information documented in German Emergency Departments. BMC Emerg Med.

[3] Hitzek J, Fischer-Rosinský A, Möckel M (2022). Influence of weekday and seasonal trends on urgency and in-hospital mortality of emergency department patients. Front Public Health.

[4] Schmiedhofer M, Möckel M, Slagman A (2016). Patient motives behind low-acuity visits to the emergency department in Germany: a qualitative study comparing urban and rural sites. BMJ Open.

[5] McCarthy ML, Zeger SL, Ding R (2009). Crowding delays treatment and lengthens emergency department length of stay, even among high-acuity patients. Ann Emerg Med.

[6] Morley C, Unwin M, Peterson GM (2018). Emergency department crowding: a systematic review of causes, consequences and solutions. PLoS One.

[7] Sartini M, Carbone A, Demartini A (2022). Overcrowding in emergency department: causes, consequences, and solutions-a narrative review. Healthcare (Basel).

[8] Blinkenberg J, Pahlavanyali S, Hetlevik Ø (2019). General practitioners’ and out-of-hours doctors’ role as gatekeeper in emergency admissions to somatic hospitals in Norway: registry-based observational study. BMC Health Serv Res.

[9] Møller TP, Ersbøll AK, Tolstrup JS (2015). Why and when citizens call for emergency help: an observational study of 211,193 medical emergency calls. Scand J Trauma Resusc Emerg Med.

[10] Van der Linden MC, Lindeboom R, van der Linden N (2014). Self-referring patients at the emergency department: appropriateness of ED use and motives for self-referral. Int J Emerg Med.

[11] (2022). Bundesministerium für Gesundheit. Das deutsche Gesundheitssystem.

[12] Baier N, Geissler A, Bech M (2019). Emergency and urgent care systems in Australia, Denmark, England, France, Germany and the Netherlands - Analyzing organization, payment and reforms. Health Policy.

[13] Fischer-Rosinský A, Slagman A, King R (2021). INDEED–utilization and cross-sectoral patterns of care for patients admitted to emergency departments in Germany: rationale and study design. Front Public Health.

[14] Mackway-Jones K, Marsden J, Windle J (2020). Ersteinschätzung in der Notaufnahme: Das Manchester–Triage–System.

[15] Gilboy N, Tanabe P, Travers D (2012). Emergency Severity Index (ESI): a triage tool for emergency department care, version 4. AHRQ Implementation handbook.

[16] Bundesinstitut für Bau-, Stadt- und Raumforschung (BBSR) (2020). INKAR - Indikatoren und Karten zur Raum- und Stadtentwicklung.

[17] Bundesinstitut für Bau-, Stadt- und Raumforschung (BBSR) (2023). Raumbeobachtung - Siedlungsstruktureller Regionstyp.

[18] Kassenärztliche Bundesvereinigung (KBV) zweistellige Fachgruppencodierung für die 8.+9. Stelle der LANR, BAR-Schlüsselverzeichnis, Anlage 35. Version 1.03.

[19] Stoica P, Selen Y (2004). Model-order selection: a review of information criterion rules. IEEE Signal Process Mag.

[20] Wickham H, Averick M, Bryan J (2019). Welcome to the tidyverse. J Open Source Softw.

[21] Bates D, Mächler M, Bolker B (2015). Fitting linear mixed-effects models using lme4. J Stat Softw.

[22] Worster A, Bledsoe RD, Cleve P (2005). Reassessing the methods of medical record review studies in emergency medicine research. Ann Emerg Med.

[23] McHale P, Wood S, Hughes K (2013). Who uses emergency departments inappropriately and when - a national cross-sectional study using a monitoring data system. BMC Med.

[24] Holzinger F, Oslislo S, Möckel M (2020). Self-referred walk-in patients in the emergency department - who and why?. BMC Health Serv Res.

[25] O’Keeffe C, Mason S, Jacques R (2018). Characterising non-urgent users of the emergency department (ED): A retrospective analysis of routine ED data. PLoS One.

[26] Kümpel L, Oslislo S, Resendiz Cantu R (2024). “I do not know the advantages of having a general practitioner” - a qualitative study exploring the views of low-acuity emergency patients without a regular general practitioner toward primary care. BMC Health Serv Res.

[27] Heinert SW, Mumford M, Kim SE (2020). User characteristics of a low-acuity emergency department alternative for low-income patients. West J Emerg Med.

[28] O’Cathain A, Foster A, Carroll C (2022). Health literacy interventions for reducing the use of primary and emergency services for minor health problems: a systematic review.

[29] Hong M, Thind A, Zaric GS (2020). The impact of improved access to after-hours primary care on emergency department and primary care utilization: a systematic review. Health Policy.

[30] Kirkland SW, Soleimani A, Rowe BH (2019). A systematic review examining the impact of redirecting low-acuity patients seeking emergency department care: is the juice worth the squeeze?. Emerg Med J.

[31] Uscher-Pines L, Pines J, Kellermann A (2013). Emergency department visits for nonurgent conditions: systematic literature review. Am J Manag Care.

[32] Somasundaram R, Geissler A, Leidel BA (2018). Beweggründe für die Inanspruchnahme von Notaufnahmen – Ergebnisse einer Patientenbefragung [Reasons for Emergency Department Visits: Results of a Patient Survey]. Gesundheitswesen.

[33] Singler K, Dormann H, Dodt C (2016). Der geriatrische Patient in der Notaufnahme. Notfall Rettungsmed.

[34] Langhoop K, Habbinga K, Greiner F, AKTIN-Notaufnahmeregister (2024). Charakteristika älterer im Vergleich zu jüngeren Notfallpatienten: Analyse von über 356.000 erfassten Besuchen des AKTIN-Notaufnahmeregisters. Med Klin Intensivmed Notfmed.

[35] Fortuna RJ, Robbins BW, Mani N (2010). Dependence on emergency care among young adults in the United States. J Gen Intern Med.

[36] Bommersbach TJ, McKean AJ, Olfson M (2023). National trends in mental health-related emergency department visits among youth, 2011–2020. JAMA.

[37] Kurian D, Sundaram V, Naidich AG (2023). Changes in low-acuity patient volume in an emergency department after launching a walk-in clinic. JACEP Open.

[38] Verband der Ersatzkassen e. V. (vdek) (2024). Daten zum Gesundheitswesen: Versicherte.

[39] Triefenbach L, Otto R, Bienzeisler J (2022). Establishing a data quality baseline in the AKTIN Emergency Department Data Registry - a secondary use perspective. Stud Health Technol Inform.

