# Supplementary material for: Prior Outpatient Care Use in Emergency Department Patients with Low- and High-acuity Conditions in Germany
Source: West J Emerg Med. 2025 Sep 20;26(5):1183–91. doi: 10.5811/westjem.38466 (PMC12591612; doi:10.5811/westjem.38466)
Supplement: Supplementary file 1 [file wjem-26-1186-s001.docx]

**Supplementary Tables and Figures – Prior outpatient care utilization in emergency department patients with low- and high-acuity conditions in Germany: a secondary data analysis**

Table 1: Methods criteria for Medical Record Review Studies in Emergency Medicine Research by Worster et al. (2005) and description on how these were adhered to in the study “Prior outpatient care utilization in emergency department patients with low- and high-acuity conditions in Germany: a secondary data analysis”.

| **Method Criterion** | **Description of criterion adherence in the study** |
| --- | --- |
| 1. Abstractors training | Data extraction was based on standardized electronic health records and statutory health insurance claims data, minimizing the need for manual abstraction. Strict data management protocols ensured consistency and reliability, eliminating the need for individual abstractor training. |
| 2. Case selection criteria | Eligible patients had at least one ED visit recorded at one of the 16 participating hospitals in 2016, were insured by a German statutory health insurance provider, and were ≥20 years old on January 1, 2016, ensuring a standardized study population. |
| 3. Variable definition | All variables were defined before data analysis to ensure consistency. The primary outcome was classified using a framework adapted from Slagman et al. (2023) based on admission status, triage category, transport type, and in-hospital mortality. Missing triage data were classified as 'not assessable'. Regional classification was defined using district codes linked to the BBSR INKAR database, while ED length of stay was restricted to a validity range of 5 minutes to 24 hours for data reliability. |
| 4. Abstraction forms | Data was extracted directly from hospital information systems and statutory health insurance claims following predefined protocols. Data harmonization ensured consistency across institutions, eliminating the need for manual abstraction forms. |
| 5. Performance monitored | Since the study relied on automated data extraction, traditional abstractor performance monitoring was not applicable. Data quality and consistency were ensured via predefined protocols, harmonization procedures, and quality control standards across data sources. |
| 6. Blind to hypothesis | Data extraction followed predefined protocols independent of the study hypothesis, ensuring objectivity in data collection and processing. |
| 7. IRR mentioned | As data abstraction was automated, interobserver reliability (IRR) assessment was not applicable. Instead, data quality was ensured through standardized protocols, harmonization procedures, and systematic validation checks. |
| 8. IRR tested | IRR testing was not applicable due to automated data extraction. Data consistency was ensured through predefined protocols, harmonization procedures, and automated quality control measures during data merging and cleaning. |
| 9. Medical record identified | The INDEED dataset integrated routine ED data from 16 German hospitals with the corresponding statutory health insurance records (2014–2017), covering 454,747 ED visits from 353,926 patients to ensure comprehensive coverage of pre- and post-ED utilization patterns. |
| 10. Sampling method | Consecutive sampling was applied to gather all eligible records for 2016 from the participating EDs to minimize selection bias. |
| 11. Missing-data management plan | Statistical methods addressed missing data. Cases missing the outcome variable were excluded, while those with missing triage data were categorized as ‘unspecified.’ Cases with missing health insurance claims data were excluded to ensure valid comparisons. Descriptive analyses were conducted to assess potential biases introduced by missing data. |
| 12. Institutional review board approval | Ethical approval was obtained from Charité - Universitätsmedizin Berlin's Ethics Committee. The data protection concept was reviewed by the TMF Working Group of Data Protection and Charité’s institutional data protection officer. Sixteen approvals from hospital data protection officers, two from federal state authorities, and eight from regulatory bodies overseeing social data usage were obtained. |

Table 2: Characteristics of adult ED visits in Germany in 2016, stratified by the included and excluded study population for the secondary data analysis “Prior outpatient care utilization in emergency department patients with low- and high-acuity conditions in Germany”. Variables include age, sex, ED presentation time, length of stay, and ED visit frequency.

|  |  |  | **Study population** | **Excluded population** | **p-value** | **Total** |
| --- | --- | --- | --- | --- | --- | --- |
|  |  |  | N = 299,914 | N = 154,833 |  | N = 454,747 |
| **Demographics** | | |  |  |  |  |
|  | Male |  | 145,284 (48.2%) | 82,422 (53.2%) | **<0.001** | 227,706 (50.1%) |
|  | Age, years (mean [SD]) | | 54 (21.1) | 55 (21.1) | **<0.001** | 54.5 (21.1) |
|  | Age, years | |  |  | **<0.001** |  |
|  |  | 18-40 | 99,481 (33.2%) | 48,522 (31.3%) |  | 148,003 (32.6%) |
|  |  | 41-60 | 76,489 (25.5%) | 40,305 (26.0%) |  | 116,794 (25.7%) |
|  |  | 61-80 | 85,481 (28.5%) | 44,938 (29.0%) |  | 130,419 (28.7%) |
|  |  | 81+ | 38,444 (12.82%) | 21,065 (13.6%) |  | 59,509 (13.1%) |
| **ED Visit** | |  |  |  |  |  |
|  | Weekday of ED presentation | |  |  | **<0.001** |  |
|  |  | Moday - Friday | 200,173 (66.7%) | 106,070 (68.5%) |  | 306,243 (67.3%) |
|  |  | Saturday / Sunday | 97,836 (32.6%) | 47,789 (30.9%) |  | 145,625 (32.0%) |
|  |  | missing | 1,905 (0.6%) | 974 (0.01%) |  | 2,879 (0.01%) |
|  | ED LOS, minutes¹ | |  |  | **<0.001** |  |
|  |  | mean (SD) | 183.1 (137.2) | 174 (124) |  | 181 (134.1) |
|  |  | median | 155 | 149 |  | 153 |
|  |  | IQR | 89-243 | 91-226 |  | 89-238 |
|  |  | NA or missing | 159,952 (53.3%) | 111,115 (71,7%) |  | 271,067 (59.6%) |
|  | ED visit frequency | |  |  | **<0.001** |  |
|  |  | 1-2 visits | 245,921 (82.0%) | 133,109 (86.0%) |  | 379,030 (83.3%) |
|  |  | 3-9 visits | 50,618 (16.9%) | 20,730 (13.4%) |  | 71,348 (15.7%) |
|  |  | >=10 visits | 3,375 (1.1%) | 994 (0.6%) |  | 4,369 (1.0%) |
| ¹includes only non-admitted patients. Abbreviations: I./km², inhabitants per square kilometer; NA, not applicable; SD, standard deviation | | | | | | |
